# Giant intradural plexiform schwannoma of the lumbosacral spine - a case report and literature review

**DOI:** 10.1186/s12891-020-03492-y

**Published:** 2020-07-11

**Authors:** Dongwoo Yu, Joon Hyuk Choi, Ikchan Jeon

**Affiliations:** 1 Department of Neurosurgery, Yeungnam University Hospital, Yeungnam University College of Medicine, 170, Hyeonchung street, Nam-Gu, Daegu, 42415 South Korea; 2 Department of Pathology, Yeungnam University Hospital, Yeungnam University College of Medicine, Daegu, South Korea

**Keywords:** Plexiform schwannoma, Spinal cord tumor, Giant, Lumbosacral

## Abstract

**Background:**

Plexiform schwannoma (PS), variant of schwannoma, often involves multiple fascicles as plexiform neurofibroma, and is usually located superficially on the dermis and subcutaneous layers. Spinal PS is extremely rare, and there is insufficient information on its natural course and treatment strategy. We describe the clinical features and treatment of giant intradural PS at the lumbosacral spine.

**Case presentation:**

A 66-year-old man presented with leg pain, paresthesia, and weakness for 2 years. Magnetic resonance imaging demonstrated a large mass lesion involving a continuous multi-lobulated bead-like mass and a cystic portion from L1 to S3. The lesion was iso-intense on T2-weighted images (WI), iso- to slightly low-intense on T1-WI, and heterogeneous enhancement on contrast-enhanced T1-WI. The large mass lesion had three portions, including a cystic mass at L1, continuous multi-lobulated bead-like mass with a cystic portion from L2 to S1, and multi-lobulated mass from S2 to S3, which were identified with severe adhesions with cauda equina on operative assessment. Grossly total extirpation was achieved at the lumbar spine, and remained three round shaped small masses at the lumbar area and a multi-lobulated round masses from S2 to S3 involving nerves related with motor function of the lower extremities and anal sphincter, respectively. Histological examination revealed multinodular or plexiform growth pattern composed of spindle-shaped tumor cells, which were diffusely and strongly positive for S100 protein with KI67 < 1%. There were no recurrence of preoperative symptoms and changes of the remained masses over a 2-year follow-up period.

**Conclusion:**

Subtotal extirpation to minimize neural deficits and close observation can be considered an appropriate treatment strategy for a giant spinal PS considering its benign prognosis and histological features, with a high risk of neurological damage during surgery.

## Background

Schwannoma is a slow-growing benign tumor arising from Schwann cells of the nerve sheaths of peripheral nerves and believed to originate from embryonic neural crest cells [1]. Generally, spinal schwannoma presents with pain and/or neurologic deficits when it grows. In some cases, it presents as a large mass that extends into the vertebra body and extraspinal space [2, 3]. Few cases of spinal giant schwannoma arising from the lumbosacral area were reported in the literature with a rare incidence [2–4]. There are still very limited data in the previous literatures. Among the schwannoma entities, approximately 5% of schwannoma develop into plexiform schwannomas (PSs) as a histological subtype [5]. PS is located superficially on the dermis and subcutaneous layers as an asymptomatic solitary nodule with slow growth in 90% of cases [6]. Non-cutaneous deep-seated lesions, particularly spinal PSs, are extremely rare, and there are still very limited data in the previous literatures [7–9].

We report the experience of surgical treatment and 2-year follow-up for the spinal giant intradural PS located extensive lumbosacral area.

## Case presentation

**Fig. 1:**
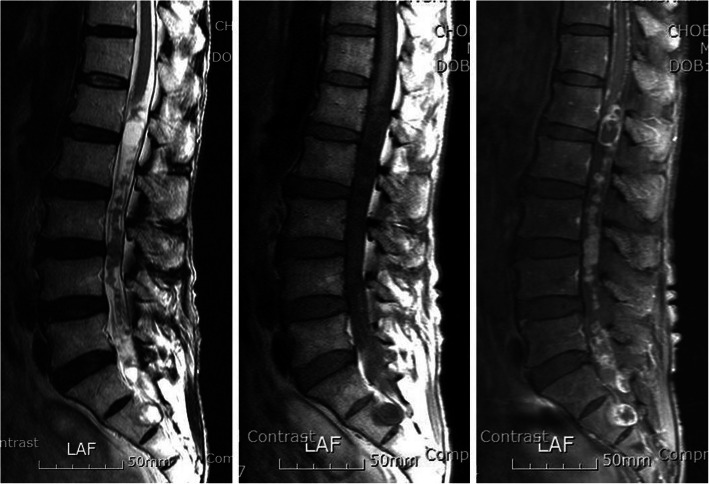
Preoperative magnetic resonance imaging shows large mass lesion with a continuous multi-lobulated bead-like mass and a cystic portion from L1 to S3. The lesion is iso-intense on T2-weighted images (WI), iso- to slightly low-intense on T1-WI, and heterogeneous enhancement on contrast-enhanced T1-WI

A 66-year-old man presented with both leg pain and paresthesia for 2 years. The symptoms aggravated in recent 6-month. The patient had a history of alcoholic liver cirrhosis and cured stomach cancer after subtotal gastrectomy 15 years ago with hematological abnormalities including thrombocytopenia (94 K) and anemia (7.6). Neurological examination showed grade 4 motor weakness of the lower extremities and neurogenic claudication with no bowel or bladder dysfunction. Magnetic resonance imaging demonstrated a large multi-lobulated bead-like mass lesion with a cystic portion from L1 to S3. The lesion was iso-intense on T2-weighted images (WI) and iso- to slightly low-intense on T1-WI with heterogeneous enhancement (Fig. 1). Neurophysiological study revealed multiple lumbar radiculopathies. There was no additional lesion of central nervous system on the whole spine and brain MR imaging with no abnormal skin lesion.

The extensive multi-lobulated mass lesions were located intraspinal canal and attached with neural components, which is judged to be difficult to access for a biopsy. We decided on surgical treatment to confirm a histological finding and improve the symptoms with removal of the mass lesion. The large mass lesion had three portions, including a cystic mass at L1, continuous multi-lobulated bead-like mass with cystic portion from L2 to S1, and multi-lobulated round masses from S2 to S3, which were identified under dural opening after total laminectomy of L1-S2. There were severe adhesions and tangles between the large mass lesion and the cauda equina with thickened arachnoid membrane and septum. The mass lesion from L1 to S1 was gross totally removed. We retained several small masses at the lumbar spine and multi-lobulated round masses from S2 to S3 involving nerves related with motor function of lower extremities and anal sphincter, respectively, under intraoperative monitoring.

**Fig. 2:**
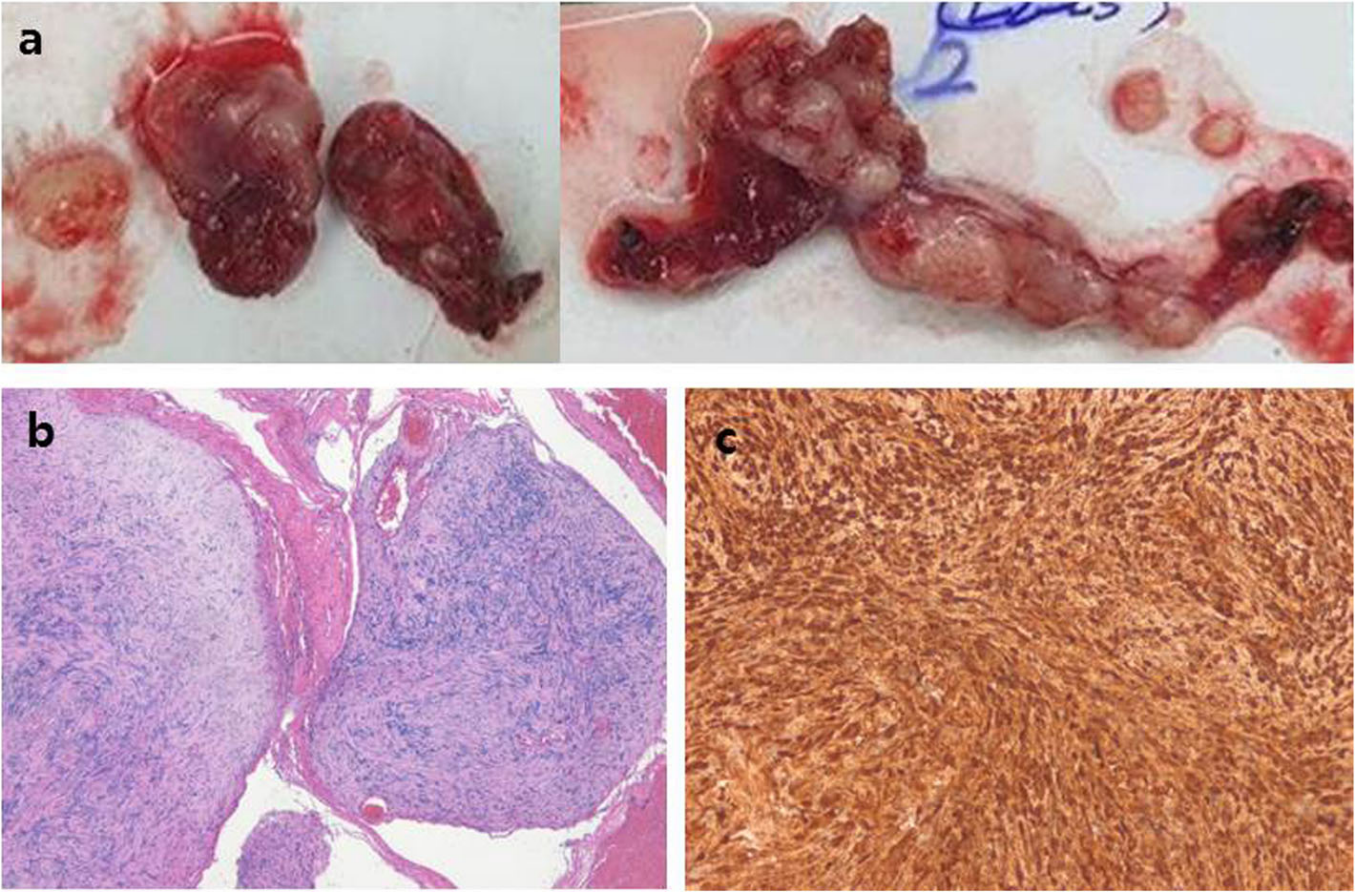
Gross and microscopic findings. There are removed two portions of the large mass lesion, including a cystic mass at L1 and continuous multi-lobulated bead-like mass with a cystic portion from L2 to S1 **(a)**. Histological examination shows multinodular growth pattern composed of nodules varying in size. The spindle-shaped tumor cells are arranged in fascicular pattern (hematoxylin-eosin, × 40) **(b)**, and diffusely and strongly positive for S100 protein (× 200) **(c)**

**Fig. 3:**
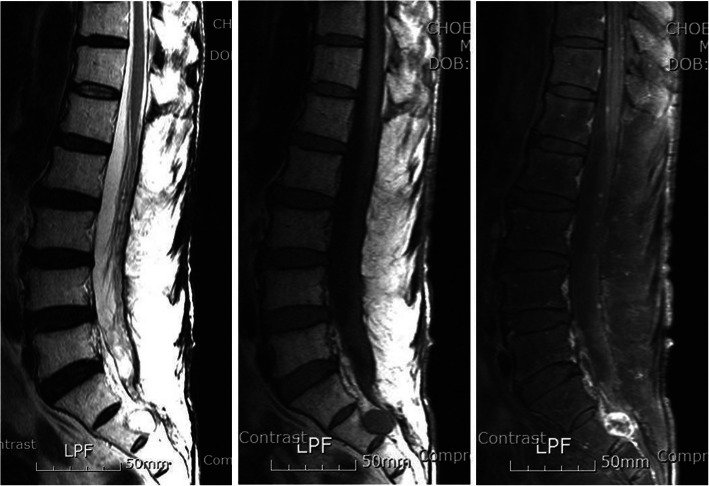
Magnetic resonance imaging at postoperative 2-year follow-up shows almost complete removal of the large mass at the lumbar spine, as well as three round small masses at the lumbar spine and a multi-lobulated round masses at S2-S3 with no changes

The patient experienced voiding difficulty with no other additional neurologic deficits after surgery. Histological examination revealed multinodular or plexiform growth pattern composed of spindle-shaped tumor cells. The tumor cells had elongated wavy nuclei and arranged in fascicular pattern with focal nuclear palisading, which were diffusely and strongly positive for S100 protein with KI67 < 1% (Fig. 2). A benign PS was eventually confirmed. We decided to observe the remaining mass lesion based on the histology and patient’s refusal for further surgery. The postoperative voiding difficulty and leg weakness completely recovered with no disturbance for normal social activities. There were almost complete removal of the large mass at the lumbar spine, as well as a multi-lobulated round masses at S2-S3 with no changes at 2-year follow-up (Fig. 3).

## Discussion

PS shows histologic features similar to those of conventional schwannoma, except for a more Antoni A pattern. However, PS shows complex growth and often involves multiple fascicles, and this differs from the single fascicle involvement of conventional schwannoma. Based on these features, it is difficult to achieve complete excision of PS without damaging multiple fascicles and neurological deficits [9]. Differential diagnosis from plexiform neurofibroma is required, which is one of the most common types of neurofibroma. Plexiform neurofibroma is related with neurofibromatosis type I and carries malignant potential risk [10]. However, unlike plexiform neurofibroma, PS is not thought to have the potential for malignant transformation [6]. There has been one report of PS, which changed to a malignant angiosarcoma [11].

Three cases of spinal giant intradural PS have been reported in the literature, two were treated with grossly total extirpation and one was treated with subtotal extirpation (Table 1). The giant spinal PS in this study was more extensive with severe adhesions and tangles between the cauda equina and mass lesion compared with previous cases in the literature. Considering the size of tumor and adhesions with neural structures, we can expect difficulty in total extirpation and risk of neural damage. We think that sometimes subtotal extirpation is more appropriate than total extirpation for spinal PS with a high risk of neural damage. It can be more clearly based on the abovementioned characteristics of a PS, including relatively benign prognosis and histological features involving multiple fascicles that could be associated with neurological deficits. In this case, there were no changes in the remaining masses at S2-S3 over a 2-year follow-up. However, there are insufficient data for spinal PS in the literature owing to its low incidence. Further studies with a large number of spinal PSs are required to demonstrate the natural course of the remaining mass lesion and identify treatment strategies in a long-term perspective.

**Table 1:** Review of literature on spinal giant intradural plexiform schwannoma

Study	Age	Sex	Site	Surgery	Follow-up	Results
Sakaura et al. [7] (2007)	16	Male	Multinodular dumbbell-shaped tumor with encroaching on the cervical cord and expanding left intervertebral foramen at C3-C4	Hemi-laminectomy and left facetectomy of C3-C4 with unilateral lateral mass screw system & Subtotal extirpation with remaining extradural tumor	1-year	Complete recovery of pain and weakness of left arm, No progression of residual tumor
Mori et al. [8] (2015)	61	Female	Intradural tumor at L2-L4	Laminectomies of L2-L4 & Total extirpation of tumor	1-year	Complete recovery of pain and weakness of both legs, No recurrence
Lam et al. [9] (2017)	65	Male	Expansible intramedullary tumor at T12-L1	Partial laminectomies of T11-L1 & Total extirpation of tumor	15-month	Improved leg weakness and remained urinary retention, No recurrence
Current study (2020)	66	Male	Large multi-lobulated bead-like mass lesion with cystic portion from L1 to S3	Laminectomies of L1-S2 & Subtotal extirpation of tumor with remaining several small masses at the lumbar spine and multi-lobulated round masses from S2 to S3	2-year	Complete recovery of pain and weakness of both legs, No recurrence

## Data Availability

The datasets during and/or analysed during the current study are available from the corresponding author on reasonable request.

## Abbreviation

PS: Plexiform schwannoma
